# Editorial Note: Olmesartan Potentiates the Anti-Angiogenic Effect of Sorafenib in Mice Bearing Ehrlich’s Ascites Carcinoma: Role of Angiotensin (1–7)

**DOI:** 10.1371/journal.pone.0316407

**Published:** 2024-12-19

**Authors:** The PLOS ONE Editors

Concerns have been raised about discrepancies in the methods and results between this article [1] and a related thesis by the first author [2]. In response, the first author stated that the discrepancies are due to errors in [2]. The first author also provided the underlying data for this article [S1–S9 Files] and additional information on the antibodies used in [1] (Table 1). The VEGFR2 antibody details were not clarified.

A member of the Editorial Board reviewed the concerns and authors’ responses, and was satisfied with the responses, but noted that hematoxylin & eosin (H&E) staining is mentioned in the Materials and Methods in [1] but is not shown.

**Table 1:** Antibody details.

Antibody	Description	Source	Catalogue number
**IGF receptors**	Mouse monoclonal antibody against human IGF-1 receptor	Thermo Fisher Scientific (Fremont, USA).	Biocare Medical CM 414 A, C
**CD31**	Mouse monoclonal antibody against human CD31	Thermo Fisher Scientific (Fremont, USA).	#MS-353-R7

## Supporting information

S1 File Individual-level quantitative data underlying the tumor weight results in [1]. (XLSX)

S2 File Individual-level quantitative data underlying the Serum VEGF results in [1]. (XLSX)

S3 File Individual-level quantitative data underlying the Serum IGF-I results in [1]. (XLSX)

S4 File Individual-level quantitative data underlying the VEGFR2 results in [1]. (XLSX)

S5 File Individual-level quantitative data underlying the IGF-IR results in [1]. (XLSX)

S6 File Individual-level quantitative data underlying the CD31 results in [1]. (XLSX)

S7 File Underlying images supporting the VEGFR results in [1]. (RAR)

S8 File Underlying images supporting the IGFR results in [1]. (RAR)

S9 File Underlying images supporting the CD31 results in [1]. (RAR)
